# Asymmetric Pseudocapacitors Based on Interfacial Engineering of Vanadium Nitride Hybrids

**DOI:** 10.3390/nano10061141

**Published:** 2020-06-10

**Authors:** Hailan Su, Tuzhi Xiong, Qirong Tan, Fang Yang, Paul B. S. Appadurai, Afeez A. Afuwape, M.-Sadeeq (Jie Tang) Balogun, Yongchao Huang, Kunkun Guo

**Affiliations:** 1 College of Materials Science and Engineering, Hunan University, Changsha 410082, China; suehai@hnu.edu.cn (H.S.); xiongtz@hnu.edu.cn (T.X.); tqr@hnu.edu.cn (Q.T.); yfang@hnu.edu.cn (F.Y.); Paul.blessington123@gmail.com (P.B.S.A.); 2 College of Computer Science and Electronic Engineering, Hunan University, Changsha 410082, China; afuwape07@hnu.edu.cn; 3 Institute of Environmental Research at Greater Bay, Key Laboratory for Water Quality and Conservation of the Pearl River Delta, Ministry of Education, Guangzhou University, Guangzhou 510006, China; huangych@gzhu.edu.cn

**Keywords:** VN nanowires, MoS_2_ nanosheets, phase boundary, pseudocapacitive charge storage, asymmetric pseudocapacitor

## Abstract

Vanadium nitride (VN) shows promising electrochemical properties as an energy storage devices electrode, specifically in supercapacitors. However, the pseudocapacitive charge storage in aqueous electrolytes shows mediocre performance. Herein, we judiciously demonstrate an impressive pseudocapacitor performance by hybridizing VN nanowires with pseudocapacitive 2D-layered MoS_2_ nanosheets. Arising from the interfacial engineering and pseudocapacitive synergistic effect between the VN and MoS_2_, the areal capacitance of VN/MoS_2_ hybrid reaches 3187.30 mF cm^−2^, which is sevenfold higher than the pristine VN (447.28 mF cm^−2^) at a current density of 2.0 mA cm^−2^. In addition, an asymmetric pseudocapacitor assembled based on VN/MoS_2_ anode and TiN coated with MnO_2_ (TiN/MnO_2_) cathode achieves a remarkable volumetric capacitance of 4.52 F cm^−3^ and energy density of 2.24 mWh cm^−3^ at a current density of 6.0 mA cm^−2^. This work opens a new opportunity for the development of high-performance electrodes in unfavorable electrolytes towards designing high areal-capacitance electrode materials for supercapacitors and beyond.

## 1. Introduction

Since Kumta’s work on vanadium nitride (VN) nanostructure [1,2], VN has extensively been used as anode material for supercapacitors (SCs) [3,4,5]. This is due to its large theoretical capacitance [6,7], suitable working negative potential window [8,9,10], excellent electrical conductivity [10] as well as pseudocapacitive properties [11]. Considering the electrochemical instability of VN in aqueous solution [12,13], the pseudocapacitance charge storage of VN that varies in different aqueous electrolytes (such as KOH and LiCl) also remains a bottleneck [14,15,16]. Recently, the storage mechanism of VN in KOH electrolyte has attracted impressive attention, while the pseudocapacitive function of VN in LiCl electrolyte is still at the infant phase. Ge at al. reported that the capacitive charge storage of VN in KOH, LiCl and LiPF_6_ are almost the same at a smaller scan rate of 5 mV s^−1^, but that of LiCl displays slightly low capacitive contribution at a high scan rate of 50 mV s^−1^ compared to those of KOH and LiPF_6_ [14]. In addition, VN is electrochemically unstable in neutral LiCl electrolytes but can be significantly improved by using LiCl/polyvinyl alcohol (PVA) gel electrolyte [17] for the design fabrication of high-performance asymmetric supercapacitors (ASC) devices. Thus, it is highly important to improve and further explore the pseudocapacitance charge storage of VN in LiCl electrolyte.

Generally, to enhance the storage performance of VN, strategies such as nanostructuring [18,19,20,21] and hybridization [22,23,24] have been employed. The hybridization approach involves carbon-based hybrids [21,25,26] and carbon-free hybrids [27,28]. For the VN-carbon-based hybrids electrodes, tremendous advancement has been realized [29,30,31], whereas carbon-free VN-based hybrids are still at their starting stage [32,33]. For example, VN could easily form composites with titanium nitride, TiN (another pseudocapacitor electrode material) [34,35] in both KOH [32] and Na_2_SO_4_ [33] electrolytes. The hybrids exhibit superior performance compared to individual VN and TiN [32,33] but the overall performance requires further improvement. Moreover, these hybrids are also rarely reported. Hybridization of two pseudocapacitor electrode materials has been reported to show attractive SC performance owing to the synergistic pseudocapacitive charge storage processes and the high theoretical capacitance of individual electrodes [36,37,38]. In addition to the sluggish progress of carbon-free VN-based hybrids, the engineering of other pseudocapacitor electrodes with VN in LiCl electrolyte is also yet to be reported. This means that carbon-free VN-based hybrids have not been reported as an electrode material for ASC devices. Therefore, tailored nanohybridization design of VN with high theoretical capacitance pseudocapacitor electrode materials is desirable not only towards boosting its pseudocapacitor storage mechanisms but also as a suitable anode material for ASC devices.

Herein, we demonstrate a synergistic hybrid electrode design, which comprises VN nanowires and MoS_2_ nanosheets (denoted VN/MoS_2_), for efficiently boosting the pseudocapacitance charge storage of VN in LiCl electrolytes. By using the as-prepared VN/MoS_2_ hybrid as anode material for SCs, its delivers a remarkably enhanced capacitance of 3187.30 F cm^−2^ at 2.0 mA cm^−2^, excellent rate capability of 1294.30 F cm^−2^ at the highest current density of 40 mA cm^−2^ as well as attractive cyclic stability with 90% capacity retention. Interestingly, by directly engaging VN/MoS_2_ as anode material in the assembly of a 2.0 V solid-state asymmetric pseudocapacitors (SSAPC) device, the assembled VN/MoS_2_//TiN/MnO_2_ SSAPC device delivers a high volumetric capacitance of 4.52 F cm^−3^, maximum energy density of 2.26 mWh cm^−3^, maximum power density of 6.01 mW cm^−3^ and cyclic stability capacity retention of 85%. These values are superior to those of pristine VN//TiN/MnO_2_, and the previously reported VN-based electrodes ASCs. The excellent performance can be attributed to the synergistic pseudocapacitance and interfacial engineering between VN and MoS_2_ as well as the individual advantage of high theoretical capacitance, which creates an opportunity for the design of VN-based negative electrodes for a high energy and power density ASC device and beyond.

## 2. Materials and Methods

### 2.1. Synthesis of VN Nanowires and VN/MoS_2_

VN was first prepared according to our previous work [12]. The first approach involves the synthesis of vanadium oxide (VO_x_) nanowires on CFC via hydrothermal method as shown in our previous works [12,39]. The second and third approaches involve annealing in NH_3_ gas and hydrothermal growth of MoS_2_, respectively. Both the second and third approaches are annealing temperature and hydrothermal time-dependent. To achieve an electrode with the best electrochemical performance, optimization of the individual active electrode remains very significant. For example, the VO_x_ was annealed in NH_3_ gas at 400, 500, 600 and 700 °C. At 400 °C, the VN was not formed (Supporting Information, Figure S1a). All reagents were purchased from Guangzhou Chemical Reagent Factory, China and directly used without further purification. An ultra-pure purification system (Master-S15Q, HHitech Instruments Co. Ltd., Shanghai, China) was used to produce 18.2 MΩ/cm water in all experiments. In a typical synthesis, 0.324 g of NH_4_VO_3_ was dissolved in 45 mL of distilled water on a magnetic hot bath at 90 °C for 10 min. After cooling at room temperature, 5 mL of ethanol (99%) was added under stirring and the pH was adjusted to ≈2 by 15 drops of concentrated HCl. A piece of the clean carbon fiber cloth, CFC (Fuel Cell Earth LLC, Woburn, MA, USA) (6.0 cm^2^) was dropped into the solution and stirred for another 5 min. The solution and the CFC were transferred to a 50 mL stainless-steel autoclave and heated in an oven at 160 °C for 12 h. After cooling, the CFC that was covered with dark-green films was washed with ethanol and distilled water several times and dried in a 60 °C oven. The covered CFC film was annealed in a tube furnace filled with NH_3_ at different temperatures of 500, 600 and 700 °C to form the VN nanowires and the samples formed were denoted VN-500, VN-600 and VN-700, respectively. VN-500 was subjected to another hydrothermal process. In a typical procedure, another mixture of 1.522 g of thiourea and 1.209 g of sodium molybdate was dissolved in 60 mL of distilled water, stirred for 15 min, transferred to a 50 mL stainless-steel autoclave and heated at 180 °C for 3, 6 and 9 h. The CFC covered with VN films was washed with distilled water and ethanol several times and the color changed to black. The samples formed denoted were VN500/3hMoS_2_ VN500/6hMoS_2_ and VN500/9hMoS_2_.

### 2.2. Electrochemical Measurements and Characterization

The cyclic voltammetry (CV) and galvanostatic charge/discharge (GCD) measurements were carried out using a CHI 760D potentiostat (Shanghai Chenhua Instrument Co. Ltd., Shanghai, China) in both the three-electrode system and asymmetrical two-electrode configurations. In a three-electrode system, a saturated Ag/AgCl and a platinum (Pt) wire were used as reference electrode and counter electrode, respectively. For electrochemical impedance spectroscopy (EIS), the amplitude signal was 5 mV, and the frequency was scanned from the 10 kHz to 5 mHz. All the electrochemical measurements were performed in a 5 M LiCl solution at room temperature. The areal capacitance (*C_A_*) of the supercapacitor was calculated from the CV curves on the basis of the following equation:

(1) $$C_{A}=\frac{S}{2\Delta VAv}$$

where *S* stands for the integral area of CV curves, *v* presents the scan rate, Δ*V* is the potential window, and *A* refers to the surface area of electrodes.

An X-ray diffractometer (D8 ADVANCE) (Bruker, Billerica, MA, USA) was used to study the phases of the samples. The samples underwent morphological analysis by using field emission scanning electron microscope (JSM-6330F) (JEOL Ltd., Akishima, Japan) and transmission electron microscopy (JEM2010-HR) (JEOL Ltd., Akishima, Japan).

### 2.3. Synthesis of Solid-State Asymmetric Pseudocapacitors (SSAPC)

The SSAPC was assembled using the VN/MoS_2_ Nanocomposite and TiN/MnO_2_ as the positive and negative electrodes, respectively and the LiCl/PVA gel as a solid electrolyte. The LiCl/PVA gel electrolyte was prepared by mixing 4.24 g LiCl and 2 g PVA in 20 mL DI water and stirred vigorously at 85 °C for 1 h. Both positive and negative electrodes were soaked in the electrolyte for 10 min and then allowed to solidify at room temperature for 6 h. The VN/MoS_2_ Nanocomposite positive and TiN/MnO_2_ negative operating potential window was set to 2.0 V.

For the SSAPC device, its volumetric capacitance, volumetric energy and volumetric power densities were calculated from the GCD curves on the basis of the following equation:

(2) $$C_{V}=\frac{I\Delta t}{V_{device}\Delta V}$$

(3) $$E=\frac{1}{2}\times C_{V}\times \Delta V^{2}$$

(4) $$P=\frac{E}{\Delta t}$$

where “*I*” represents the discharge current, Δ*t* presents the discharge time, Δ*V* is the potential window, and *V_device_* stands for the volumetric capacitance of the device. The area and thickness of the SSAPC device are 1.0 cm^2^ and 0.08 cm, respectively.

## 3. Results and Discussion

### 3.1. Characterizations

VN/MoS_2_ hybrids were prepared of carbon fiber cloth (CFC) through a three-step approach. X-ray diffraction (XRD) analysis for the VN samples obtained at temperatures of 500, 600 and 700 °C, shows three peaks that correspond to cubic VN (Joint Committee on Powder Diffraction Standards, JCPDS-#35-0768) and are denoted as VN-500, VN-600 and VN-700 (Figure S1a). The difference between these three VN samples is that the intensity of the (111), (200) and (220) phases of VN increases with increasing temperature indicating that the crystallinity of VN increases upon increasing temperature. The scanning electron microscopy (SEM) analyses of VN-500, VN-600 and VN-700 revealed that the morphology of the samples is nanowires and temperatures did not show any significant effect on the nanowires (Figure S2). The electrochemical properties of the VN electrodes were tested as shown in Figure S3 and VN-500 exhibits the optimum storage performance.

**Figure 1:**
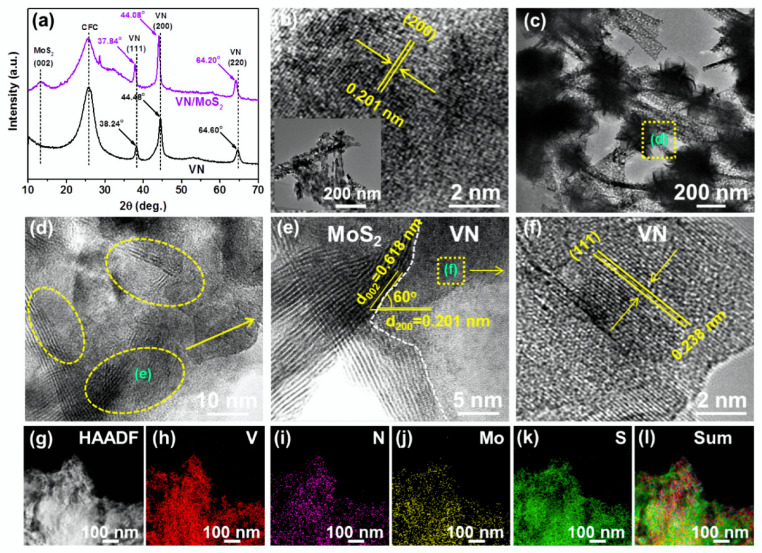
(**a**) XRD spectra of vanadium nitride (VN) and VN/MoS_2_. (**b**) High-resolution TEM (HRTEM) image of VN. Inset is the low magnification TEM image of VN. (**c**) TEM image of VN/MoS_2_. (**d**) HRTEM image of VN/MoS_2_ showing different phase boundaries of VN/MoS_2_. (**e**) HRTEM image obtained from “d” identifying the lattice spacings and the angular mismatch between the phases of VN and MoS_2_. (**f**) HTEM collected from “e” showing another VN phase. (**g**–**l**) EDS elemental mapping showing the distribution of each element in the hybrids.

To form the hybrid, MoS_2_ was grown at the VN-500 grown on CFC as a substrate/current collector. Our attempt to improve the pseudocapacitive charge storage mechanism of VN in LiCl electrolyte is by decorating VN with another active electrode that allows rapid flow and transfer of Li-ion and electrons, as well as high capacitance. A typical example is MoS_2_ [40]. MoS_2_ is a pseudocapacitor electrode material that shows higher intrinsic ionic conductivity than metal oxides [41,42] and its multivalence states are beneficial for higher energy density [43]. The capacitance of MoS_2_ nanostructures originates from its surface redox activity, which accounts for higher pseudocapacitance at a higher rate capability [44]. Moreover, MoS_2_ and other dichalcogenides have previously been proved to have a significantly active site on metal nitride surfaces, which is highly favorable for capacity enhancement and structural stability [45,46]. Therefore, the hydrothermal growth period of MoS_2_ on the VN-500 substrate was controlled at 3 h, 6 h and 9 h and the obtained samples are denoted VN500/3hMoS_2_, VN500/6hMoS_2_ and VN500/9hMoS_2_. The detailed XRD, SEM and electrochemical properties can be found in Figures S1b, S4 and S5, respectively, with VN500/6hMoS_2_ emerging as the optimized sample. Further characterizations were observed on VN-500 (denoted VN) and VN500/6hMoS_2_ (denoted VN/MoS_2_). By comparing VN with VN/MoS_2_, the XRD spectrum of VN/MoS_2_ in Figure 1a reveals the presence of both VN and MoS_2_ (hexagonal molybdenite-2H MoS_2_, JCPDS-#37-1492) phases, confirming the formation of the hybrid. Moreover, XRD spectra further show that the three main VN peaks in the VN samples are shifted to a lower theta degree with 0.40° after hybridization of MoS_2_. This shift can be related to the electronic interaction between VN and MoS_2_, and can also lead to rapid ion and electron transfer towards achieving excellent storage performance [47,48]. It should be noted that the main peak attributed to the CFC at 25.8° did not exhibit any shift in theta degree, indicating that the hybridization process shows no effect on the sample phases.

Further morphological characterization on the VN and VN/MoS_2_ was studied via transmission electron microscopy (TEM). The TEM image in Figure 1b inset consists of interlaced nanowires, which are well-ascribed to previously reported VN nanowires [13,49]. High-resolution TEM (HRTEM) collected at the edge of the nanowires exhibits well-resolved lattice fringes of 0.201 nm, which corresponds to the (200) plane of cubic VN (Figure 1b, JCPDS-#35-0768). For comparison, the nanosheet morphology of MoS_2_ uniformly coated on CFC is shown in Figure S6. The HRTEM image in Figure S6d indicates the 2D layer structure of MoS_2_ with an interlayer lattice distance of 0.617 nm, corresponding to the (002) plane of MoS_2_ and also affirming the formation of MoS_2_ (JCPDS-#37-1492). For the hybrid sample, after the second hydrothermal process, nanosheets of MoS_2_ were uniformly coated on the surface of VN interlaced nanowires (Figure 1c). HRTEM collected at the edge of VN/MoS_2_ further clarify that both the phases of VN and MoS_2_ share a lot of interface boundaries (Figure 1d). By enlarging the HRTEM image in Figure 1d, the phase boundaries between MoS_2_ and VN were clearly observed. The interplanar distances of 0.618 nm and 0.201 nm were both observed at the MoS_2_ region and VN region of the phase boundaries, respectively (Figure 1e). The boundaries between VN and MoS_2_ marked an angular mismatch of 62° between the (002) phase of MoS_2_ and the (200) plane of VN. In lithium-ion batteries, lattice interfaces and angular mismatches usually lead to more active site formation serving as an additional lithium storage capacity [50,51]. In addition, the interfaces between MoS_2_ and other catalysts could enhance the HER performance of the hybrid catalyst due to the electronic interaction that arises from the interfaces [52,53]. According to Figure 1f, a lattice spacing of 0.238 nm corresponding to (111) plane of cubic VN was also revealed from Figure 1e. According to the results obtained for SEM and TEM analyses, the composite formation of VN and MoS_2_ exhibits a high capacitance with good cycle performance and remarkable rate capability and also beneficial for structural stability [54,55,56]. Hence, we hypothesize that the phase boundaries between VN and MoS_2_ in our hybrid could also lead to capacitance enhancement. Besides, energy dispersive X-ray spectroscopy (EDS) elemental mapping further exposes the distribution of the elements V, N, Mo and S (Figure 1g–l). All these results justify the formation of the VN/MoS_2_ hybrid sample.

### 3.2. Electrochemical Properties

**Figure 2:**
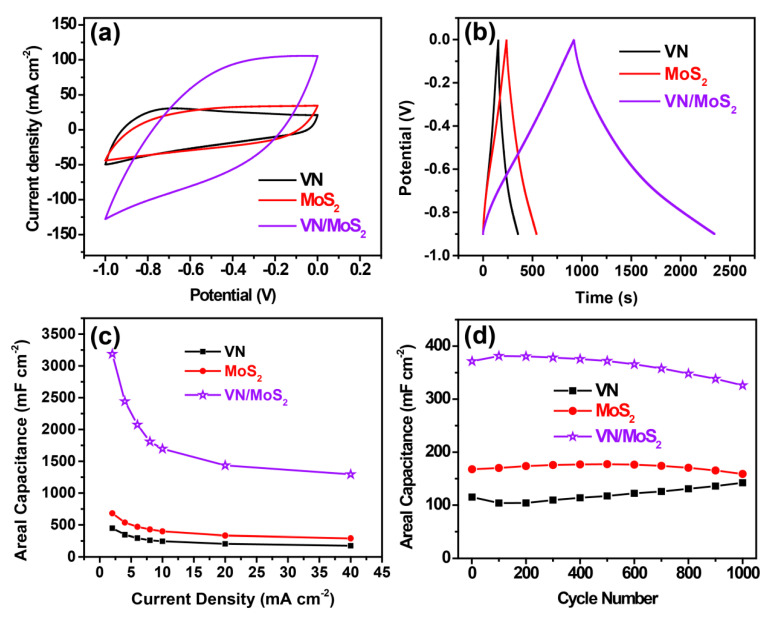
Electrochemical properties. (**a**) Cyclic voltammetry (CV) curves at a scan rate of 100 mV s^−1^, (**b**) galvanostatic charge/discharge profiles at a current density of 2 mA cm^−2^, (**c**) areal capacitance obtained from the Galvanostatic charge/discharge profiles as a function of current density and (**d**) Cycling performance collected at a scan rate of 100 mV/s of VN, MoS_2_ and VN/MoS_2_ electrodes.

The electrochemical properties of VN and VN/MoS_2_ were studied and depicted in Figure 2. MoS_2_ was also introduced for comparison and proper discussion. According to Figure 2a, the cyclic voltammetry curve (CV) of VN/MoS_2_ hybrid displays a higher current density than the VN and MoS_2_ electrodes, demonstrating significant performance enhancement due to hybridization. This indicates that VN/MoS_2_ shows the highest electrochemical surface area and activity. More importantly, in addition, the CV curves of the hybrid at various scan rates are still quasilinear in shapes and also maintained superior electrochemical surface areas and current densities (Figure S7a–c), indicating higher pseudocapacitive properties and high-rate performance. Indeed, the areal capacitances of VN/MoS_2_ is 1487.28 mF cm^−2^ at the scan rate of 5 mV s^−1^ and 267.11 mF cm^−2^ at 200 mV s^−1^, which is substantially higher than that of its counterparts, pristine VN (386.44 and 134.71 mF cm^−2^) and MoS_2_ (527.07 and 158.40 mF cm^−2^), revealing the superior and excellent rate performance of hybrid electrodes (Figure S7d). The galvanostatic charge–discharge measurements of the three electrodes were studied at different current densities ranging from 2.0 to 40.0 mA cm^−2^ as shown in Figure S8. Compared to VN and MoS_2_ electrodes, VN/MoS_2_ electrode exhibits a longer discharge time but a shorter charge time at the current density of 2.0 mA cm^−2^ (Figure 2b) indicating improved capacitance and its suitability for commercial applications. More importantly, the areal capacitance determined based on the galvanostatic profiles of the VN/MoS_2_ electrode reaches 3187.30 mF cm^−2^ at the current density of 2.0 mA cm^−2^. The result is sevenfold higher than VN (447.28 mF cm^−2^), 4.5-fold higher than MoS_2_ (447.28 mF cm^−2^) (Figure 2c) and recently reported VN-based electrodes such as VNQDs/PC hybrid (1124.0 mF cm^−2^ @ 4 mA cm^−2^), [57] VN/CNTF (564.0 mF cm^−2^ @ 1.0 mA cm^−2^) [58], MVN@NC NWs film (282.0 mF cm^−2^ @ 1.44 mA cm^−2^) [59] etc.

At different current densities of 4, 6, 8, 10, 20 and 40 mA cm^−2^, the areal capacitance of VN/MoS_2_ hybrids are 2442.02, 2074.61, 1808.84, 1696.40, 1435.05 and 1294.30 mF cm^−2^. The results are still dramatically higher than VN nanowires and MoS_2_ nanosheets, and are also the best among the preceding works on VN-based electrodes (Table S1) [12,60,61]. Such outstanding performance can be associated with the synergistic response as a result of the interfacial engineering between VN and MoS_2_ that is beneficial for capacitance enhancement. These results are in agreement with the morphological analyses. The cyclic stability performance tests of the three electrodes were tested at constant scan rates of 100 mV s^−1^ for 1000 cycles and compared in Figure 2d. VN/MoS_2_ still maintained higher areal capacitance that its counterparts, retaining 88% of its initial capacitance at 371.48 mF cm^−2^. The high areal capacitance of our hybrid electrode is also the best when compared to other previously reported related electrodes (Table S1) [56,60,61]. After the cyclic stability test, the CV curve and the shape of VN/MoS_2_ electrode and XRD spectra are almost the same as the initial one, suggesting excellent structural and phase stability, respectively (Figure S9). The CV, galvanostatic charge–discharge and cyclic stability profiles sufficiently justify that the hybridization of MoS_2_ on VN can significantly improve the electrochemical properties of VN nanowires electrode.

### 3.3. Capacitive Storage Mechanism

Pseudocapacitor electrode materials could exhibit high rates performance due to the relatively high electrical conductivity of some of these electrodes [62,63]. To explore more information on the electrode conductivity, we first examined the internal resistance of the electrodes to gain insight into the electrode kinetics. The corresponding *iR*-drop (where *i* and *R* represent the current and resistance, respectively) of VN/MoS_2_ is much smaller than VN at low current densities (between 2.0–10.0 mA cm^−2^) but is almost the same at high current densities (Figure S10a). Moreover, based on the electrochemical impedance spectroscopy (EIS) results, the Nyquist plots show that the charge transfer resistance, *R_ct_* (i.e., the semi-circle at the low-frequency region) of the hybrid and pristine VN samples are almost the same, indicating that the integration of MoS_2_ may not necessarily enhance the hybrid electrode conductivity (Figure S10c). However, the Warburg resistance (*W_s_*) that is related to the diffusion of ions in VN/MoS_2_ cells is closer to 90° than the VN and MoS_2_ cells at a higher frequency region (Figure S10b) [49,64]. Such favorable ion diffusion in VN/MoS_2_ can be related to MoS_2_ incorporation that possesses the ability to facilitate diffusion of Li ions during electrochemical processes [45].

As discussed above, both VN and MoS_2_ are pseudocapacitor electrode materials. It should be pointed out that pseudocapacitance can hardly exist in most materials at the bulk state level but can occur in two conditions: (i) during the design of hybrid electrode materials to form a monolithic single material, slow charge-transfer kinetics could occur from the low electrical conductivity of one of the electroactive materials [65]. The “slow charge-transfer kinetics” is assessed by comparing the VN/MoS_2_ hybrid with pristine VN nanowires. As it is well known that VN possesses better electrical conductivity [12] than MoS_2_, the formation of VN/MoS_2_ composites may lead to “slow charge-transfer kinetics”. However, VN faces the problem of electrochemically instability in aqueous solutions, which causes severe capacitance loss during cycling process, and further requires improvement. Nonetheless, MoS_2_ possess high ionic conductivity and high theoretical capacitance of 1504 F g^−1^ [66]. However, its poor conductivity and limited exposure of edge sites also leads to low specific capacitance. Nonetheless, MoS_2_ usually exhibits excellent performance when directly grown on the conductive surfaces [67] just like the growth of our MoS_2_ on binder-free conductive VN nanowires. Therefore, the growth of MoS_2_ on VN nanowires is an advantage. (ii) Extrinsic pseudocapacitance is possible due to the appearance of the materials in nanoscale with high surface area as well as an enhanced electrode/electrolyte contact area [68,69]. In our case, the enlarged surface area and shortened diffusion distance of VN/MoS_2_ may lead to rapid kinetics, which causes higher capacitive current contribution. We further discussed the pseudocapacitive mechanism of VN/MoS_2_ system. Firstly, The V ions of VN show a wide range of oxidation states (V^2+^ to V^5+^) leading to its pseudocapacitive behavior [1,2]. In addition, MoS_2_ consists of three atom layers (S-Mo-S) stacking coupled with weak van der Waals interaction and its Mo ions show a series of oxidation states ranging from Mo^2+^ to Mo^6+^. The wide range of oxidation states causes its pseudocapacitance abilities. Thus, electrode materials with various oxidation states usually utilize their redox reaction at the interface or heterojunctions for pseudocapacitive mechanism. Such a phenomenon also occurs in our VN/MoS_2_ hybrid system. In our case, the enlarged surface area and shortened diffusion distance of VN/MoS_2_ may lead to rapid kinetics, which causes higher capacitive current contribution. The combination of VN nanowires and MoS_2_ nanosheets creates effective phase interfaces between the electrodes and also provide rapid transportation of ions for the surface redox reactions [70] due to the excellent ionic conductivity of MoS_2_. Such an approach enables easy diffusion of ions and higher pseudocapacitance is achieved in VN/MoS_2_ compared to pristine VN and MoS_2_. Thus, our pseudocapacitance contribution data is based on the above mentioned conditions. To verify this mechanism, the pseudocapacitance contribution was calculated from the CV curves of individual VN, MoS_2_ and VN/MoS_2_ electrodes.

**Figure 3:**
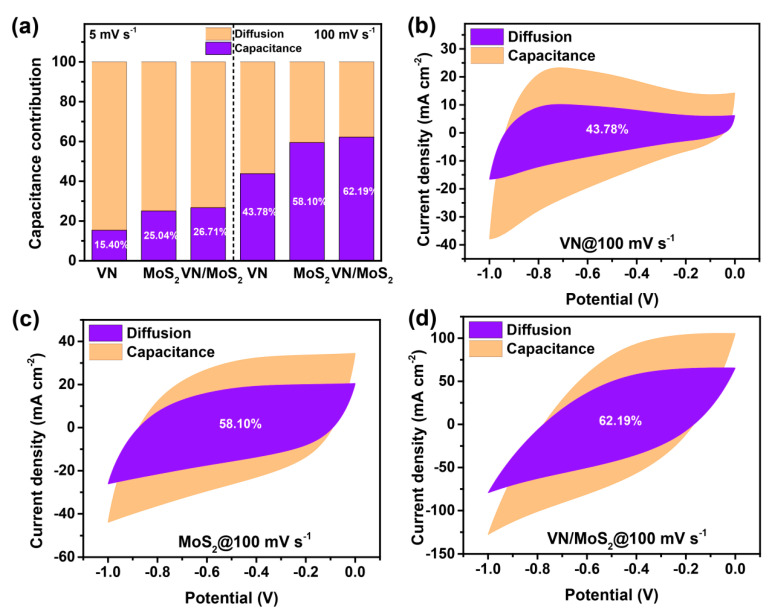
Pseudocapacitive Charge Storage Mechanism. (**a**) Capacitive and diffusion-controlled capacitance contribution of VN and VN/MoS_2_ at scan rates of 5 and 100 mV s^−1^. CV curves showing the capacitive- and diffusion-controlled contributions at 100 mV s^−1^ of (**b**) VN, (**c**) MoS_2_ and (**d**) VN/MoS_2_.

Previous literature have unveiled the pseudocapacitive charge storage mechanism of VN in a KOH electrolyte [14,15,16] but the pseudocapacitive charge storage mechanism of VN in the LiCl electrolyte attracts less focus. Ge’s group explored the pseudocapacitive charge storage of VN in LiCl electrolytes and reported the poor pseudocapacitance charge of VN in LiCl compared to KOH electrolytes. However, it is very essential to improve the capacitance of VN in an LiCl electrolyte towards achieving high-performance solid-state-supercapacitors (SSCs). As a result of the excellent rate capability of VN/MoS_2_ hybrid electrode, the pseudocapacitive charge storage mechanisms of the three electrodes were analyzed from their corresponding CV profiles in Figure S7. To understand the pseudocapacitive behavior during electrochemical storage, we determined the capacitive- and diffusion-controlled capacitance. The relationship between these two controlled capacities can be expressed by the Equation (1) [71]:

*I* (*V*) = *k*_1_*v* + *k*_2_*v*^1/2^ (5)

where the capacitive and diffusion contribution is *k*_1_ and *k*_2_, respectively, and *v* is the scan rate. At a low scan rate of 5 mV s^−1^, the CV curves in Figure S11a shows that the current density of VN/MoS_2_ is also undoubtedly higher than the current density of VN and MoS_2_. At low scan rate of 5 mV s^−^^1^, the capacitive contribution of VN is the smallest (Figure S11b) but increases in MoS_2_ (Figure S11c) but is the highest in the hybrid (Figure S11d). The result indicates that the harmonization of MoS_2_ with VN has the positive effect of enhancing the storage mechanism of VN. At a high scan rate of 100 mV s^−1^, the capacitive contribution of VN electrode is not up to half of the total capacitance (Figure 3b), affirming the poor pseudocapacitive storage mechanism of VN (Figure 3a). However, upon the combination with MoS_2_, the as-prepared VN/MoS_2_ hybrid reaches a maximum capacitive contribution (Figure 3d), significantly higher than that of pristine MoS_2_ (Figure 3c) and further validating and establishing the enhanced pseudocapacitive storage mechanism of VN in LiCl electrolytes through MoS_2_ introduction.

**Scheme 1:**
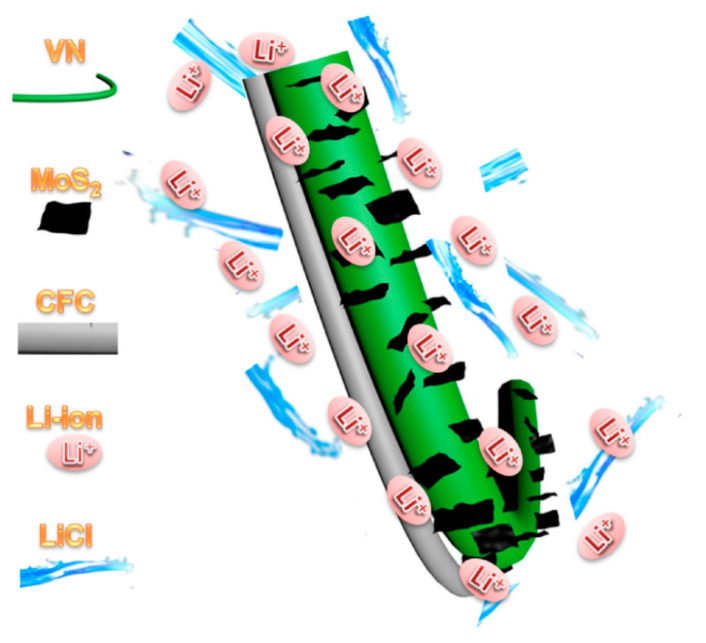
Schematic representation of the VN/MoS_2_ providing Li-ion and electron channels.

The excellent pseudocapacitance charge storage mechanism and the overall improvement in the CFT-VN@MoS_2_ electrode system can also be explained using the schematic diagram in Scheme 1.

The synergistic effects in the electrode 3D network created benign interactions at the boundaries of 1D VN and 2D MoS_2_ that meant that an Li ion from the electrolyte could easily penetrate the electrode network without any external back-up. The integration of the MoS_2_ increases flexibility of ions and electrons flow, and impressively hastens the transportation of Li ions. MoS_2_ incorporation did not really show a specific contribution to the whole hybrid electrical conductivity but effectively synergizes ion diffusion, which enhances the electrode kinetics and outstanding rate performance were achieved. Hence, the incorporation of MoS_2_ drastically contributed to the pseudocapacitance storage mechanism of VN leading to high and excellent capacitance. The CFC displays no capacitance contribution to the electrode (ref) but constitutes a 3D channel that enables smooth migration of electrons and ions, and further facilitates direct connection with VN interlaced nanowires. Furthermore, it has been already justified that phase boundaries between the lattice fringes of two electrodes constructing an angular mismatch are avenues for accommodating additional Li ion [72,73,74]. The interfaces between the (200) plane of VN and (002) plane of MoS_2_ will generate additional storage capacitance, also leading to high capacitance of the synergistic VN/MoS_2_ hybrid.

### 3.4. Solid-State Asymmetric Pseudocapacitor (SSAPC) Device Performance

**Figure 4:**
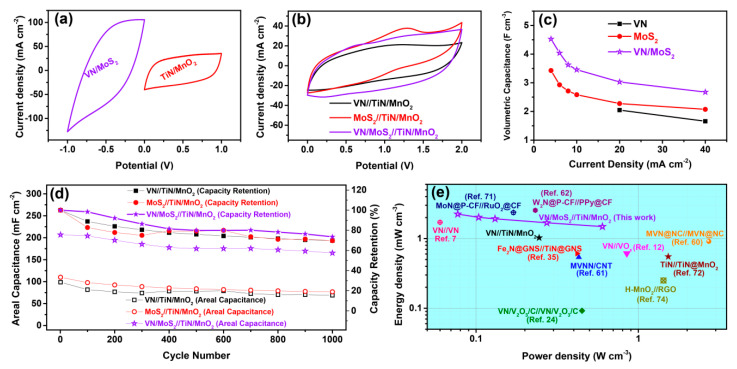
(**a**) CV curves of VN/MoS_2_ and TiN/MnO_2_ anode and cathode collected at a scan rate of 100 mV s^−1^. (**b**) CV curves of VN//TiN/MnO_2_, MoS_2_//TiN/MnO_2_ and VN/MoS_2_//TiN/MnO_2_–solid-state asymmetric pseudocapacitor (SSAPC) devices at a scan rate of 100 mV s^−1^. (**c**) Volumetric capacitance of VN//TiN/MnO_2_ and VN/MoS_2_//TiN/MnO_2_ as a function of current density. (**d**) Cyclic performance and capacitance retention of VN//TiN/MnO_2_ and VN/MoS_2_//TiN/MnO_2_-SSAPC. (**e**) Ragone plots of VN/MoS_2_//TiN/MnO_2_ device with the energy and power densities of other asymmetric supercapacitor (ASC) devices included for comparison.

Having boosted the pseudocapacitive storage performance of VN nanowires using MoS_2_ incorporation in LiCl electrolytes, it is quite convenient to assemble a solid-state asymmetric pseudocapacitor device (SSAPC) by utilizing a VN/MoS_2_ hybrid electrode as anode and the cathode material selected was MnO_2_ coated on TiN (denoted TiN/MnO_2_). Both TiN [35,75,76] and MnO_2_ [77,78,79] are also pseudocapacitor electrode materials. The cathode performance of TiN/MnO_2_ is shown in Figure S12. In order to achieve optimal storage performance, the charge between the negative and positive electrodes requires balancing before cell assembling. As seen in Figure 4a, the ratio of the as-prepared VN/MoS_2_ anode to TiN@MnO_2_ cathode was determined to be 1:0.5. The SSAPCs were assembled using LiCl-PVA gel as the electrolyte. Pristine VN was also assembled for comparison.

Figure 4b presents the CV curves of VN//TiN/MnO_2_, MoS_2_//TiN/MnO_2_ and VN/MoS_2_//TiN/MnO_2_. As expected, VN/MoS_2_//TiN/MnO_2_ deliver a current density higher than those of VN//TiN/MnO_2_ and VN/MoS_2_//TiN/MnO_2_, confirming the superior performance of the hybrid device. The quasilinear curves were also observed for the three devices at scan rates and their electrochemical potential reaches 2.0 V (Figure S13). However, the galvanostatic charge–discharge measurements reveal that VN//TiN/MnO_2_ could not charge/discharge beyond 20 mA cm^−2^ from 40 mA cm^−2^ (Figure S14a), while VN/MoS_2_//TiN/MnO_2_ device charges and discharges up to 6.0 mA cm^−2^ from 40 mA cm^−2^ (Figure S14b), indicating that our as-assembled VN/MoS_2_//TiN/MnO_2_ device exhibits excellent pseudocapacitive behavior. MoS_2_//TiN/MnO_2_ device could also charge and discharge up to 6.0 mA cm^−2^ from 40 mA cm^−2^ (Figure S14c) but shows less discharging period compared to VN/MoS_2_//TiN/MnO_2_. Based on the current densities of the three devices, the maximum volumetric capacitance (based on the volume of the entire device) of VN/MoS_2_//TiN/MnO_2_ reaches 4.52 F cm^−3^ at a current density of 4.0 mA cm^−2^ and could maintain a volumetric capacitance of 2.67 F cm^−3^ at a current density of 40 mA cm^−2^, while that of VN//TiN/MnO_2_ is 1.64 F cm^−3^ at 40 mA cm^−2^ and VN/MoS_2_//TiN/MnO_2_ is 2.07 F cm^−3^ at 40 mA cm^−2^ (Figure 4c). The results confirmed the excellent rate capability of the VN/MoS_2_//TiN/MnO_2_ device as significantly higher than some of the previously reported VN-based and other ASC devices [35,60,61,80,81] (Table S2). The stability performance of VN/MoS_2_//TiN/MnO_2_ device achieves capacity retention of 75% (165.16 mF cm^−2^ by capacitance), while VN//TiN/MnO_2_ is only left with 69% (76.82 mF cm^−2^ by capacitance) and/MoS_2_//TiN/MnO_2_ is only left with 69% (68.85 mF cm^−2^ by capacitance) after 1000 cycles (Figure 4d).

Finally, in Figure 4e, we compare the volumetric energy and power densities of the VN/MoS_2_//TiN/MnO_2_ device with the VN//TiN/MnO_2_ device and the devices that have previously been reported. A detailed calculation on the volumetric energy and power densities can be found in the experimental section. Our as-assembled VN/MoS_2_//TiN/MnO_2_ SSAPC device conveys maximum volumetric energy density of 2.24 mWh cm^−3^ at current density of 6.0 mA cm^−2^ (minimum volumetric power density of 0.07 W cm^−3^), outperforming its counterpart, VN/TiN/MnO_2_ and recently reported VN-based, metal nitride-based and non-metal nitride-based devices, such as VN//VO_x_ (0.61 mWh cm^−3^, 0.5 mA cm^−2^) [12], MVNN/CNT//MVNN/CNT (0.54 mWh cm^−3^, 0.025 mA cm^−^^2^) [61], VN/V_2_O_3_/C//VN/V_2_O_3_/C (0.092 cm Wh m^−2^, 0.5 mA cm^−2^) [24], Fe_2_N@GNS//TiN@GNS (0.61 mWh cm^−3^, 2 A g^−1^) [35], TiN//TiN@MnO_2_ (0.55 mWh cm^−3^, 1.0 mA cm^−2^) [81], TiN//TiN (0.05 mWh cm^−3^, 2.5 mA cm^−3^) [82], H-MnO_2_//RGO (0.25 @ 2.0 mA cm^−2^) [83], 3DHPC-NiCo_2_S_4_//3DHPC-Fe_2_O_3_ [84], and so on. In addition, the VN/MoS_2_//TiN/MnO_2_ SSAPC device also achieved a maximum volumetric power density of 0.61 W cm^−3^ (minimum volumetric energy density of 1.48 mWh cm^−3^) at a current density of 40 mA cm^−2^, which is not only superior to its VN//TiN/MnO_2_ counterpart (1.02 W cm^−3^ at a current density of 6.0 mA cm^−2^) but is also superior to previously reported VN- and metal nitride-based devices such as W_2_N@P-CF//PPy@CF (0.232 @ 20.0 mA cm^−2^) [56], MoN@P-CF//RuO_2_@CF (0.17 @ 16.0 mA cm^−2^) [80], as well as some other non-metal nitride ASCs such as 3DHPC-NiCo_2_S_4_//3DHPC-Fe_2_O_3_ [84], CNT//ZnCo_2_O_4_@NiMoO_4_·H_2_O [85], and RuO_2_//Ti_3_C_2_T_x_ [54] (Table S2).

## 4. Conclusions

In conclusion, we have manifested that the integration of MoS_2_ nanosheets can successfully enhance the pseudocapacitance charge storage of VN nanowires. Morphological analysis revealed that there exist phase boundaries between VN and MoS_2_ that could lead to additional storage capacitance in the VN/MoS_2_. In comparison with highly conductivity VN nanowires, the as-prepared VN/MoS_2_ achieves a high areal capacitance of 3187.30 mF cm^−2^ at 2.0 mA cm^−2^, which is dramatically superior to that of pristine VN (447.28 mF cm^−2^) and excellent rate capability with an areal capacitance of 1294.30 mF cm^−2^ at 40.0 mA cm^−2^ (better than that of VN at 175.36 mF cm^−2^). More importantly, VN/MoS_2_ exhibits remarkable improvement towards pseudocapacitance charge storage in LiCl electrolytes. The capacitive contribution at 100 mV s^−1^ increases from 43.78% in VN electrode to 62.19% after hybridizing VN with MoS_2_ (VN/MoS_2_ hybrid). The exceptional performance of the as-prepared hybrid is attributed to the synergistic pseudocapacitance contributions from VN and MoS_2_, as well as the phase boundaries between the lattice spacings of both VN and MoS_2_. Finally, an SSAPC device assembled based on VN/MoS_2_ anode and TiN/MnO_2_ cathode achieved a maximum volumetric energy density of 2.24 mWh cm^−3^ at a current density of 6.0 mA cm^−2^ and maximum volumetric power density of 0.60 mWh cm^−3^ at a current density of 40.0 mA cm^−2^. This work did not only demonstrate the first VN-based pseudocapacitors as a high volumetric energy and power anode for ASCs but also deciphers the pseudocapacitance storage mechanism of VN in a LiCl electrolyte.

## Supplementary Materials

The following are available online at https://www.mdpi.com/2079-4991/10/6/1141/s1, Figure S1. (a) XRD spectra of VOx samples annealed at different temperatures ranging from 400–700 °C. (b). XRD spectra of MoS_2_ hydrothermally grown on VN-500 at different hydrothermal duration of 3 h, 6 h and 9 h; Figure S2. SEM images of (a,b) VM-500, (c,d) VN-600 and (e,f) VN-700. Figure S3. Electrochemical properties of VN-500, VN-600 and VN-700; Figure S4. SEM images of (a,b) VN500/3hMoS_2_, (c,d) VN500/6hMoS_2_ and (e,f) VN500/9hMoS_2_; Figure S5. Electrochemical properties of VN500/3hMoS_2_, VN500/6hMoS_2_ and VN500/9hMoS_2_; Figure S6. (a) XRD spectra, (b,c) SEM images and (d,e) TEM images of MoS2; Figure S7. CV curves of (a) VN and (b) VN/MoS_2_. (c) Rate performance of VN and VN/MoS_2_ electrodes; Figure S8. Galvanostatic charge/discharge profiles of (a) VN and (b) VN/MoS_2_ electrodes; Figure S9. (a) CV curves and (b) XRD spectra of VN/MoS_2_ electrodes before and after cyclic stability test; Figure S10. (a) iR drop, and (b,c) Nyquist plot of VN and VN/MoS_2_ electrodes; Figure S11. Electrochemical properties of TiN/MnO_2_. (a) CV curves at different scan rates, (b) Galvanostatic charge/discharge profiles at different current densities, (c) Nyquist plot and (d) Rate performance as a function of scan rates; Figure S12. CV curves of (a) VN/MoS_2_//TiN/MnO_2_ and (b) VN//TiN/MnO_2_ at different scan rates. (c) Galvanostatic charge/discharge profiles of VN//TiN/MnO_2_ device at current densities of 20.0 and 40.0 mA cm^−2^. (d) The volumetric capacitance of VN//TiN/MnO_2_ and VN/MoS_2_//TiN/MnO_2_ as a function of the scan rate; Figure S13. Electrochemical properties of MoS_2_ compared with VN. (a) iR drop and (b) Nyquist plot, (c) CV curves at scan rates of 100 mV s^−1^, (d) Capacitive and diffusion-controlled capacitance contribution at scan rates of 5 and 100 mV s^−1^, (e) Galvanostatic charge/discharge profiles at a current density of 2.00 mA cm^−2^ and (f) Rate performance of VN and MoS_2_ electrodes; Figure S14. Galvanostatic charge–discharge curves of the (a) VN//TiN/MnO_2_, (b) MoS_2_//TiN/MnO_2_ and (c) VN/MoS_2_//TiN/MnO_2_-SSAPC devices obtained at different current densities up to voltage window of 2.0 V.; Table S1. Comparison of the areal capacitance of VN-based electrodes in different electrolytes; Table S2. Comparison of the VN-based and other related PVA-based solid-state supercapacitor devices.
