# Supplementary material for: Asymmetric Pseudocapacitors Based on Interfacial Engineering of Vanadium Nitride Hybrids
Source: Nanomaterials (Basel). 2020 Jun 10;10(6):1141. doi: 10.3390/nano10061141 (PMC7353334; doi:10.3390/nano10061141)
Supplement: Supplementary file 1 [file nanomaterials-10-01141-s001.pdf]

Supporting Information

Asymmetric Pseudocapacitors Based on Interfacial Engineering of Vanadium Nitride Hybrids

Hailan Su [1], Tuzhi Xiong [1], Qirong Tan [1], Fang Yang [1], Paul B. S. Appadurai [1], Afeez A. Afuwape [2], M.-Sadeeq (Jie Tang) Balogun [1,*] Yongchao Huang[3], and Kunkun Guo [1,*]

[1] College of Materials Science and Engineering, Hunan University, Changsha 410082, Hunan, China; suehai@hnu.edu.cn (H.S.); xiongtz@hnu.edu.cn (T.X.); tqr@hnu.edu.cn (Q.T.); yfang@hnu.edu.cn (F.Y.); Paul.blessington123@gmail.com (P.B.S.A.)

[2] College of Computer Science and Electronic Engineering, Hunan University, Changsha 410082, Hunan, China; afuwape07@hnu.edu.cn

[3] Institute of Environmental Research at Greater Bay, Key Laboratory for Water Quality and Conservation of the Pearl River Delta, Ministry of Education, Guangzhou University, Guangzhou, 510006, China; huangych@gzhu.edu.cn

* Correspondence: balogun@hnu.edu.cn (M.-S. (J.T.) B.); kunkunguo@hnu.edu.cn (K.G.)

Experimental Section Cont.

Synthesis of TiN/MnO_2 on CFC. Firstly, TiO_2 nanowires were grown on carbon cloth by a seed-assisted hydrothermal method. The carbon cloth (5.0×3.0 cm) was cleaned with ethanol and then dried at 60 ℃. Then, the carbon cloth was immersed into a mixture of 0.43 mL $TiCl_4$ and 20 mL distilled water. After 2 min, the carbon cloth was blow-dried and heated on a hot-plate in the air at 300 ℃, repeat this step three times, resulting in TiO_2 nanoparticles on the carbon cloth surface. 0.6 mL of titanium n-butoxide was added into a mixed solution of 20 mL concentrated hydrochloric acid (37 %) and 20 mL distilled water, and then stirred into a transparent solution. The obtained solution together with the above-mentioned carbon cloth was transferred into a 50 mL Teflon-lined stainless steel autoclave, and heated in an electric oven at 160 ℃ for 5 hours. After naturally cooled down to room temperature, the carbon cloth was thoroughly washed with DI water and dried. Subsequently, TiO_2 nanorods were conversed to TiN nanorods through thermal treatment at 800 ℃ in NH_3 (200 cc min^{-1}) for 1 h. MnO_2 was grown on a TiN substrate via electrodeposition method as following: the carbon cloth with TiN NWs (2.0 ×0.5 cm) was conducted in a three-electrode cell with carbon cloth with TiN NWs as working electrode, Pt wire, and Ag/AgCl as counter electrode and reference electrode, respectively. The electrodeposition was carried out in a mixed solution of 0.1 M $Mn(Ac)_2$ and 0.1 M Na_2SO_4 with 1.0 V constant potential for 90 s.

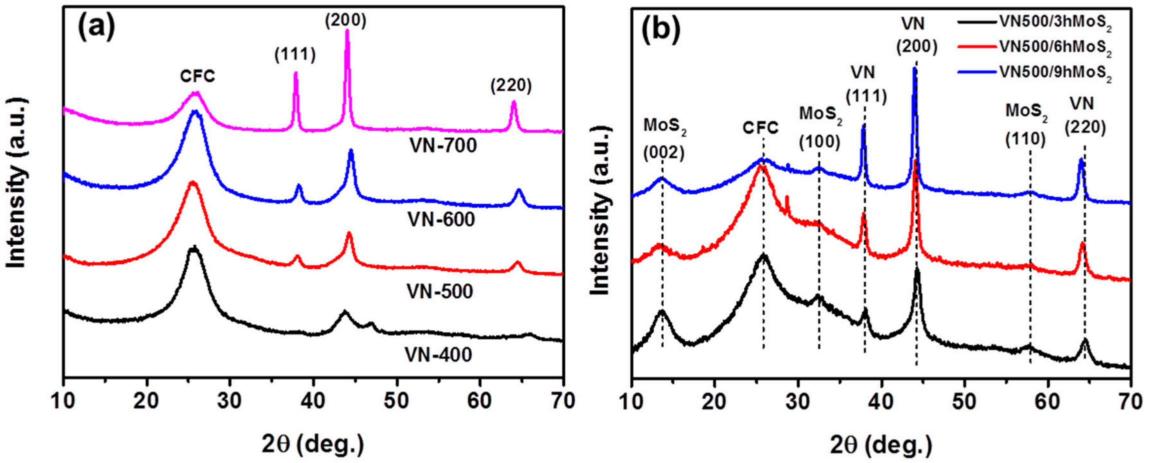

Figure S1. (a) XRD spectra of VO_x samples annealed at different temperatures ranging from 400-700 ^{o}C. (b). XRD spectra of MoS_2 hydrothermally grown on VN-500 at different hydrothermal duration of 3 h, 6 h and 9 h. Upon the increase in the hydrothermal period of MoS_2, the intensity of VN peaks gradually reduces, while those peaks intensity corresponding to hexagonal molybdenite-2H MoS_2 (JCPDS-#37-1492) increases accordingly (Figure S1b).

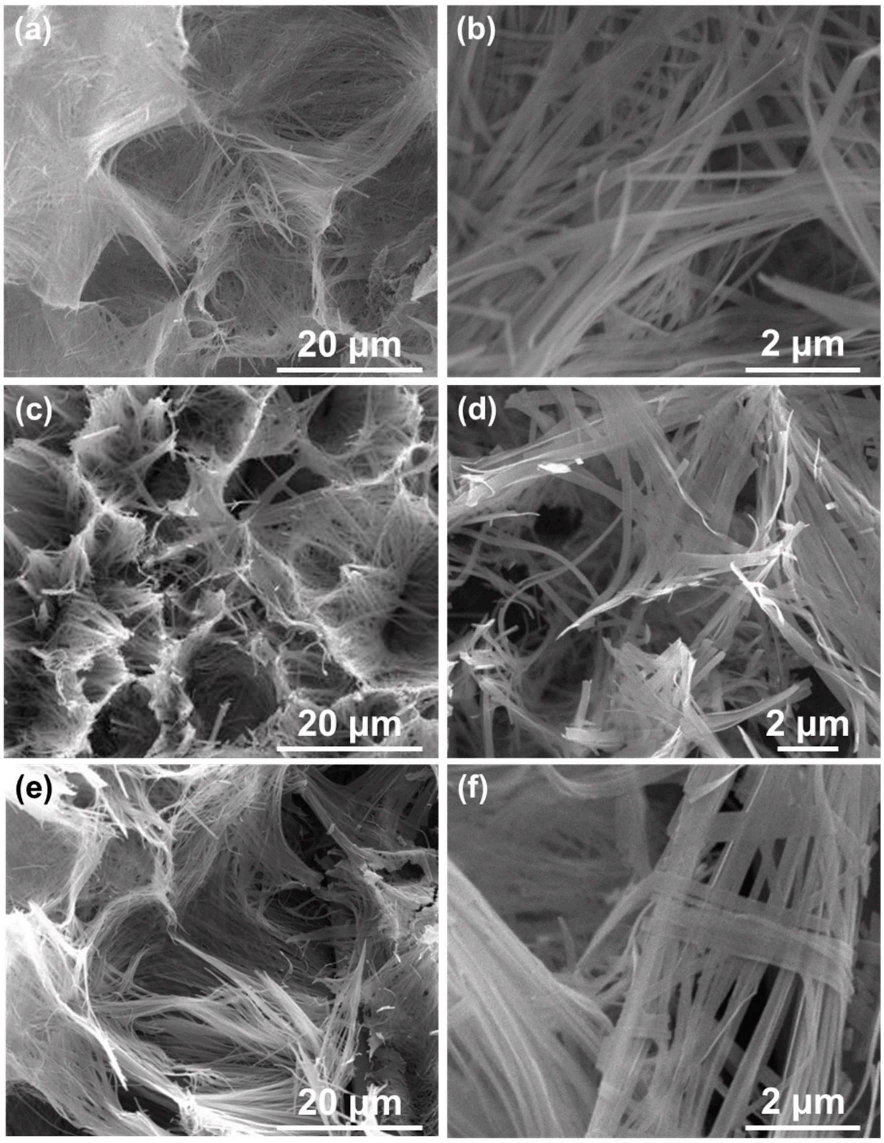

Figure S2. SEM images of (a, b) VM-500, (c, d) VN-600 and (e, f) VN-700.

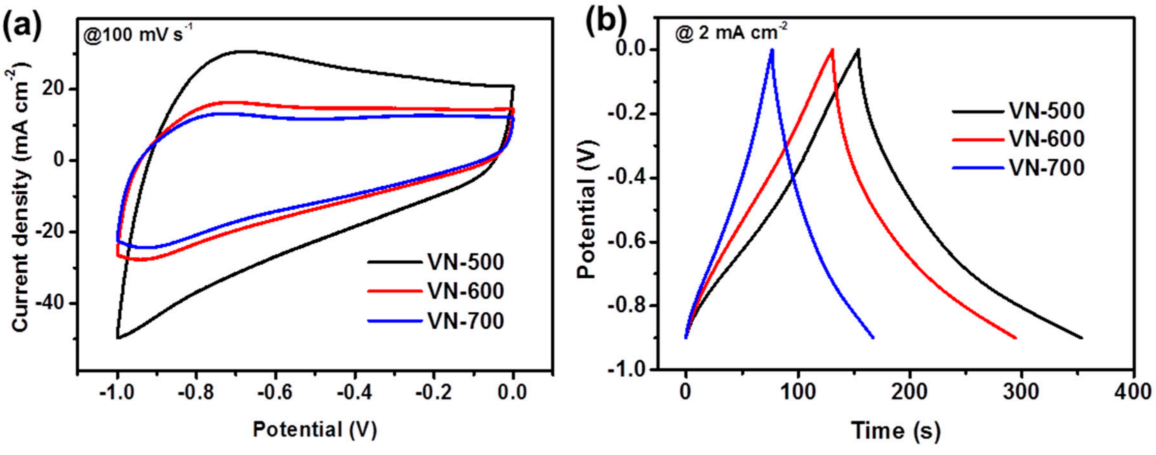

Figure S3. Electrochemical properties of VN-500, VN-600 and VN-700.

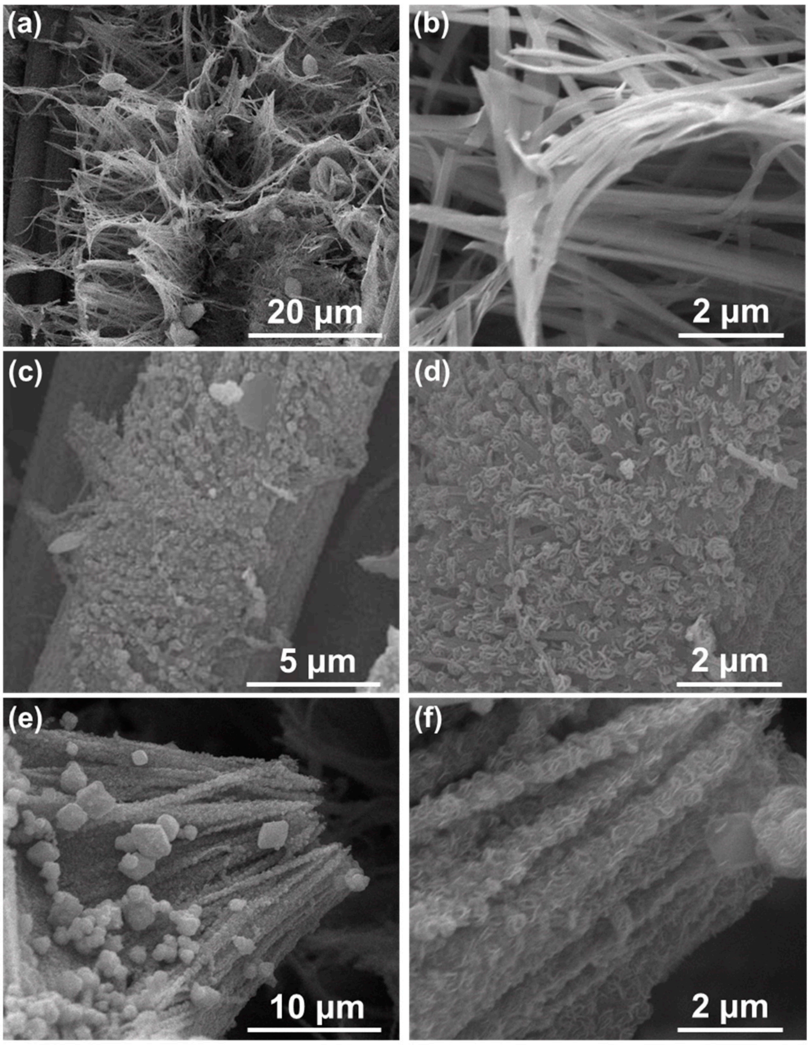

Figure S4. SEM images of (a, b) VN500/3hMoS$_2$, (c, d) VN500/6hMoS$_2$ and (e, f) VN500/9hMoS$_2$. SEM images of the hybrid samples revealed some differences, with VN500/9hMoS$_2$ displaying MoS$_2$ nanosheets coated on the VN-500 nanowires (Figure S4e and S4f), and much more concentrated than those of VN500/3hMoS$_2$ (Figure S4a and S4b) and VN500/6hMoS$_2$ (Figure S4c and S4d).

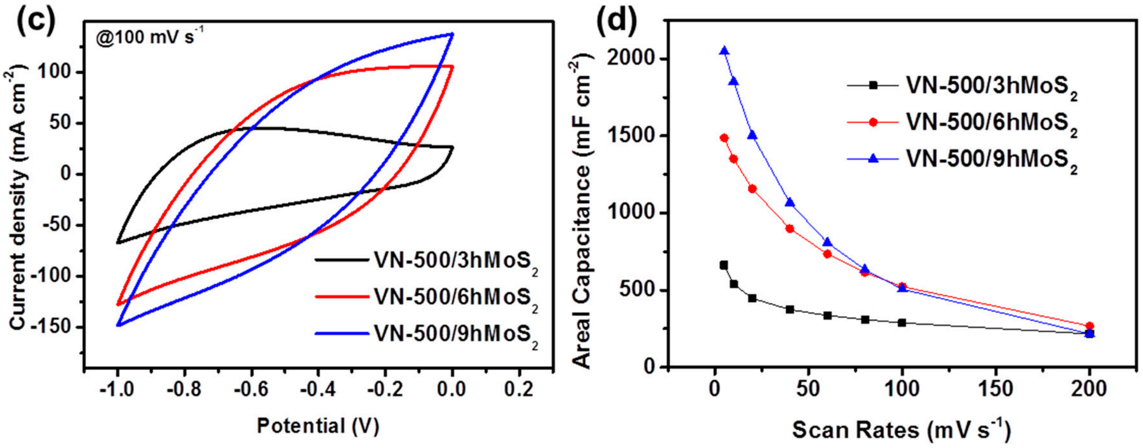

Figure S5. Electrochemical properties of VN500/3hMoS_2, VN500/6hMoS_2 and VN500/9hMoS_2. Electrochemical properties displayed in Figure S5 also revealed that the performance of both VN500/6hMoS_2 and VN500/9hMoS_2 are very similar (VN500/9hMoS_2 slightly higher at low scan rates but VN500/6hMoS_2 show better performance at higher scan rate implying better rate performance) but significantly better than that of VN500/3hMoS_2. Hence, we select VN500/6hMoS_2 as the optimized VN/MoS_2 hybrid due to the economic factor of time and electricity consumed during hydrothermal to 9 h.

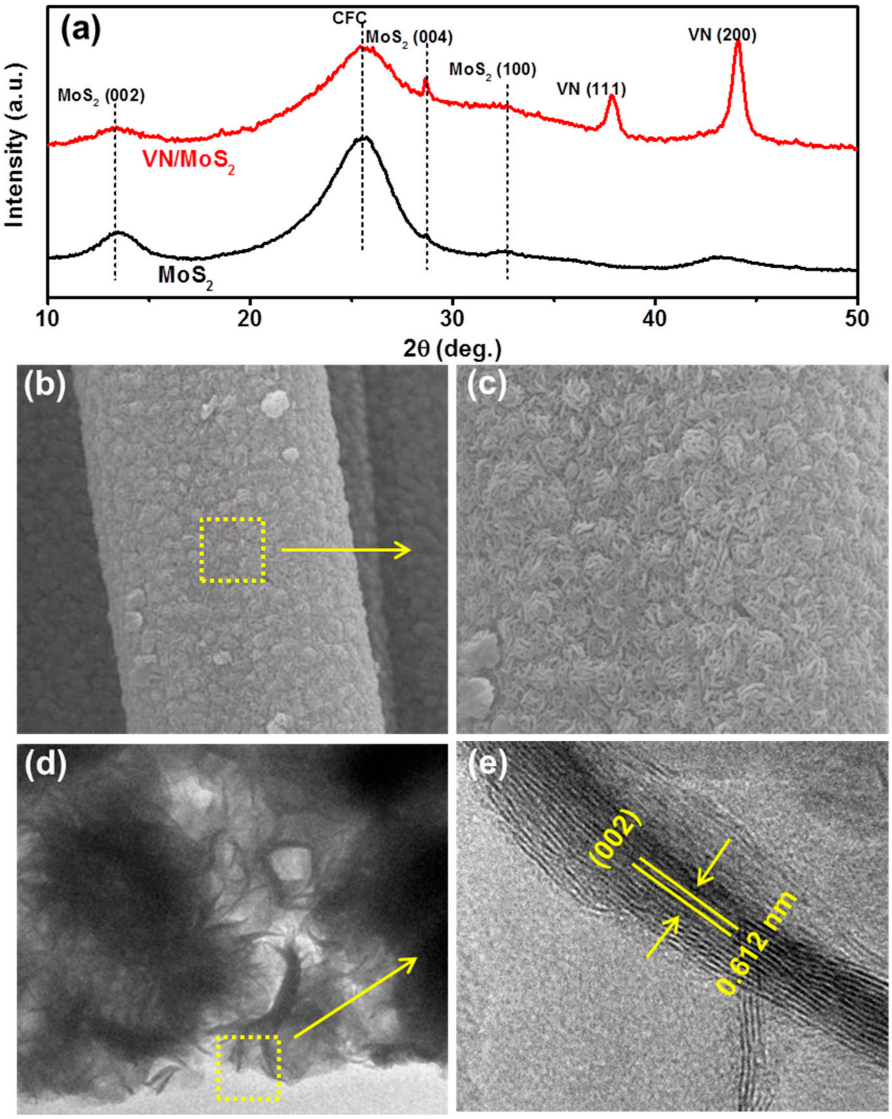

Figure S6. (a) XRD spectra, (b, c) SEM images and (d, e) TEM images of MoS_2.

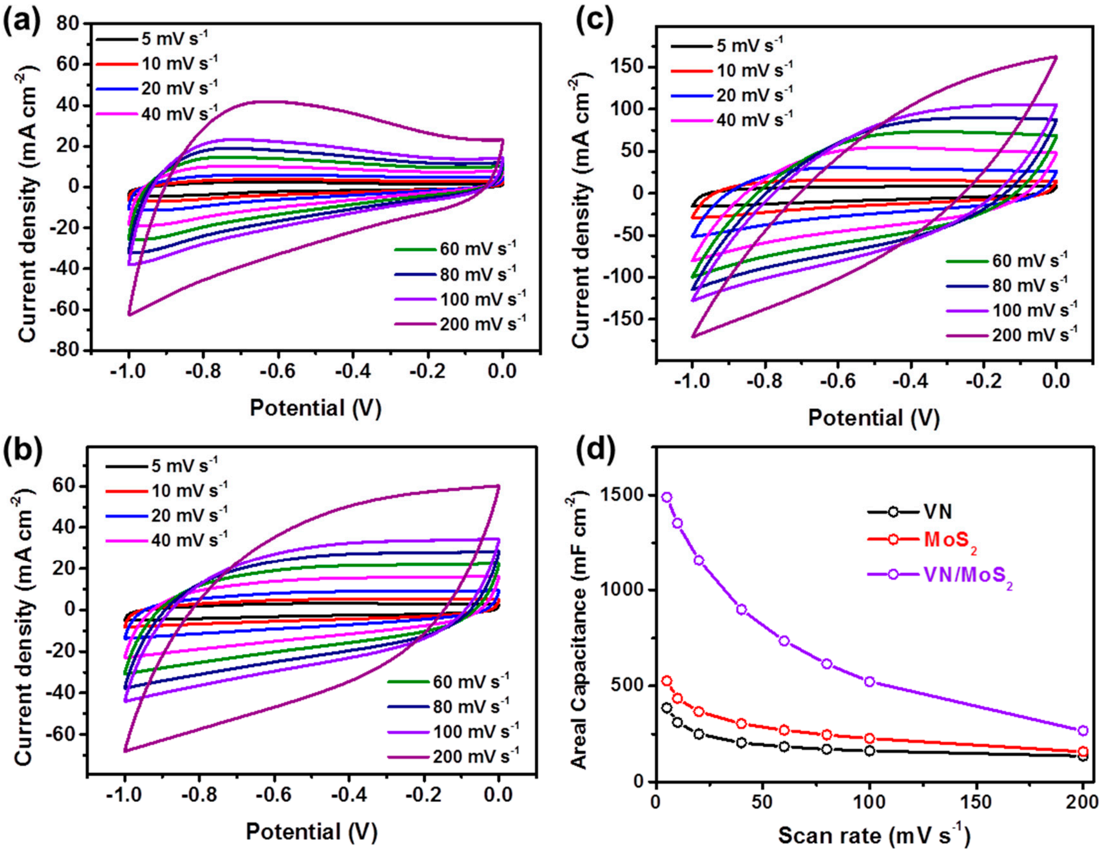

Figure S7. CV curves of (a) VN, (b) MoS_2 and (c) VN/MoS_2. (d) Rate performance of VN and VN/MoS_2 electrodes.

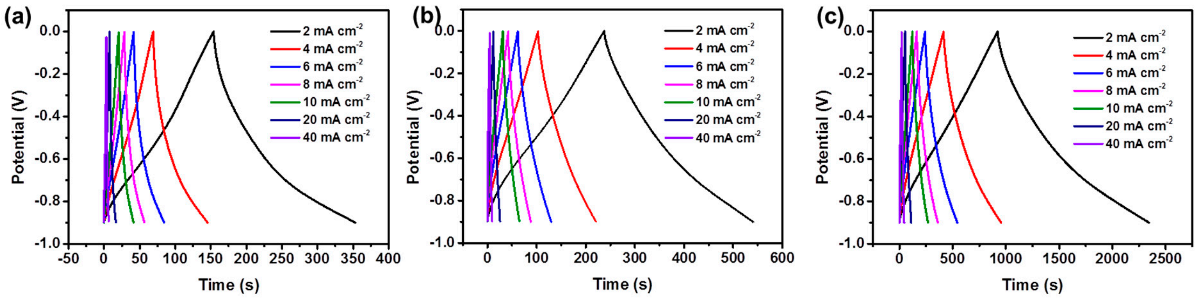

Figure S8. Galvanostatic charge/discharge profiles of (a) VN, (b) MoS_2 and (c) VN/MoS_2 electrodes.

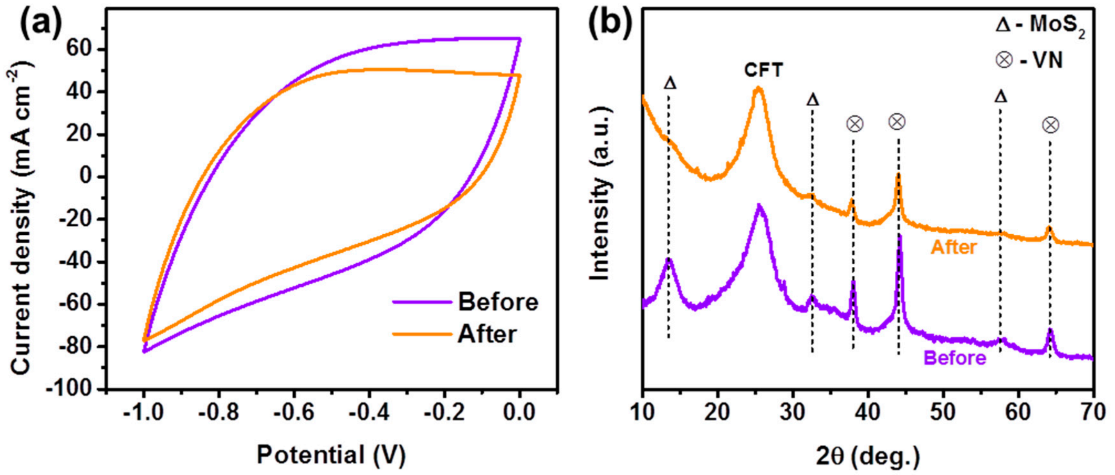

Figure S9. (a) CV curves and (b) XRD spectra of VN/MoS_2 electrodes before and after cyclic stability test.

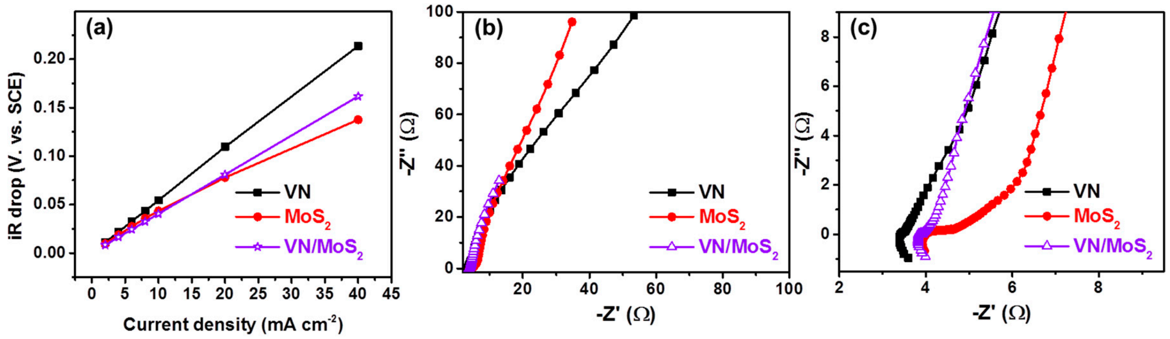

Figure S10. (a) *iR* drop, and (b, c) Nyquist plot of VN, MoS_2 and VN/MoS_2 electrodes.

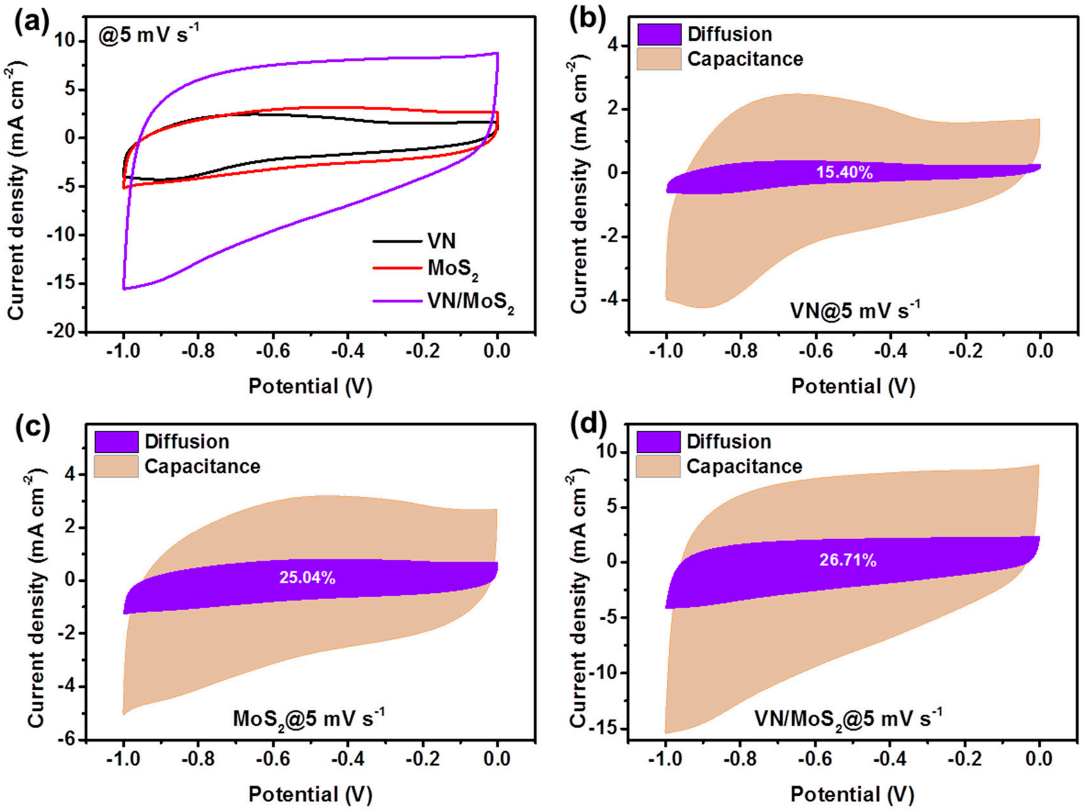

Figure S11. Pseudocapacitive Charge Storage Mechanism. (a) CV curves of VN and VN/MoS_2 electrodes at a scan rate of 5 mV s^{-1}. CV curves showing the capacitive and diffusion-controlled contributions at 5 mV s^{-1} of (b) VN, (c) MoS_2 and (d) VN/MoS_2.

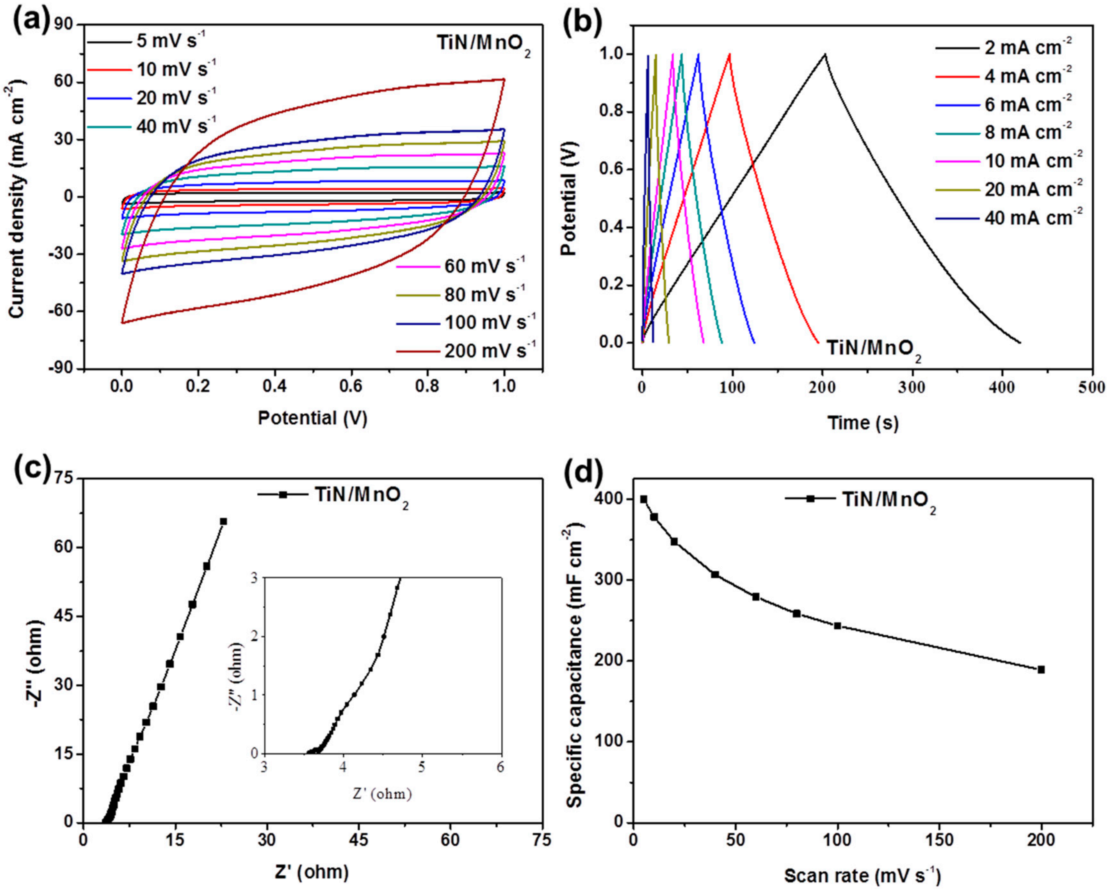

Figure S12. Electrochemical properties of TiN/MnO_2. (a) CV curves at different scan rates, (b) Galvanostatic charge/discharge profiles at a different current densities, (c) Nyquist plot and (d) Rate performance as a function of scan rates.

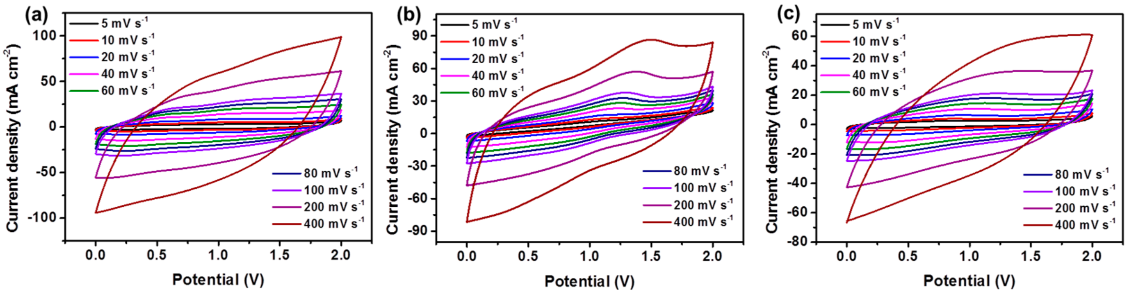

Figure S13. CV curves of (a) VN/MoS_2//TiN/MnO_2, (b) VN//TiN/MnO_2 and (c) MoS_2//TiN/MnO_2 at different scan rates.

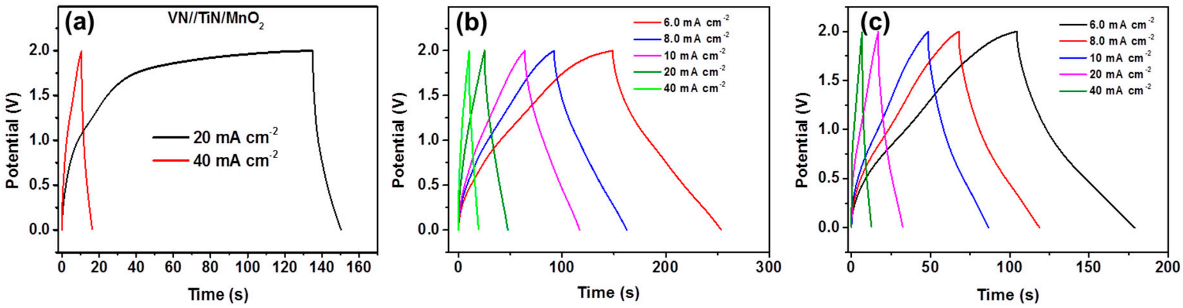

Figure S14. Galvanostatic charge–discharge curves of the (a) VN//TiN/MnO_2, (b) MoS_2//TiN/MnO_2 and (c) VN/MoS_2//TiN/MnO_2-SSAPC devices obtained at different current densities up to voltage window of 2.0 V.

Table S1. Comparison of the areal capacitance of VN-based electrodes in different electrolytes.

Electrodes	Electrolyte	Areal Capacitance (mF cm^{-2})	Rate Performance (mF cm^{-2})
VN/MoS_2 (This work)	5 M LiCl	3187.30 @ 2.0 mA cm^{-2}	1294.30 @ 40.0 mA cm^{-2}
VN nanowires (This work)	5 M LiCl	447.28 @ 2.0 mA cm^{-2}	175.36 @ 40.0 mA cm^{-2}
Mesoporous VN/CNT [1]	H_3PO_4	178.0 @ 1.1 mA cm^{-2}	≈ 75 @ 11.0 mA cm^{-2}
VNQDs/PC hybrid [2]	6 M KOH	1124.0 @ 4 mA cm^{-2}	209 @ 17.0 mA cm^{-2}
MVN@NC NWs film [3]	6 M KOH	282.0 @ 1.44 mA cm^{-2}	200 @ 30.0 mA cm^{-2}
VN/CNTF [4]	6 M KOH	564.0 @ 1.0 mA cm^{-2}	361 @ 10.0 mA cm^{-2}
VN thin films [3]	6 M KOH	238.2 @ 5 mV s^{-1}	24.7 @ 100 mV s^{-1}

Table S2. Comparison of the VN-based and other related PVA-based solid-state supercapacitor devices.

Devices	Electrolyte	Maximum Energy Density (mWh cm^{-3})	Maximum Power Density (W cm^{-3})
$VN/MoS_2//TiN/MnO_2$ This work	LiCl/PVA	2.24 @ 6.0 mA cm^{-2}	0.60 @ 40.0 mA cm^{-2}
$VN//TiN/MnO_2$ This work	LiCl/PVA	0.240 @ 6.0 mA cm^{-2}	0.92 @ 40.0 mA cm^{-2}
MVNN/CNT//MVNN/CNT [1]	H_3PO_4/PVA	0.54 @ 0.025 mA cm^{-3}	0.43 @ 0.5 mA cm^{-3}
$VN//VO_x$ [5]	LiCl/PVA	0.61 mWh cm^{-3}	0.85 W cm^{-3}
VN//VN Nanofiber [6]	LiCl/PVA	0.89 m Wh cm^{-3}	0.016 m W cm^{-3}
MVN@NC//MVN@NC NWs[7]	PVA/PAAS/KOH	0.97 @ 0.051 mA cm^{-3}	2.72 @ 0.408 mA cm^{-3}
$VN/V_2O_3/C//VN/V_2O_3/C$ [8]	Na_2SO_4	0.092 cm Wh m^{-2} @ 0.5 mA cm^{-2}	0.45 W cm^{-2} @ 10 mA cm^{-2}
MoN@P-CF//RuO_2@CF [9]	H_2SO_4/PVA	2.36 @ 4.0 mA cm^{-2}	0.17 @ 16.0 mA cm^{-2}
Fe_2N@GNS//TiN@GNS[10]	LiCl/PVA	0.61 @ 2.0 A g^{-1}	0.42 @ 16.0 A g^{-1}
W_2N@P-CF//PPy@CF[11]	H_2SO_4	2.54 @ 4.0 mA cm^{-2}	0.232 @ 20.0 mA cm^{-2}
TiN//TiN@MnO_2[12]	LiCl/PVA	0.55 @ 1.0 mA cm^{-2}	1.53 @ 8.0 mA cm^{-2}
H-MnO_2//RGO [13]	LiCl/PVA	0.25 @ 2.0 mA cm^{-2}	1.43 @ 12.0 mA cm^{-2}
3DHPC-$NiCo_2S_4$//3DHPC-Fe_2O_3 [14]	PVA	1.71 @ 2.0 A g^{-1}	0.06 @ 10.0 A g^{-1}

References

1. Xiao, X.; Peng, X.; Jin, H.; Li, T.; Zhang, C.; Gao, B.; Hu, B.; Huo, K.; Zhou, J. Freestanding mesoporous VN/CNT hybrid electrodes for flexible all-solid-state supercapacitors. *Adv. Mater.* **2013**, *25*, 5091-5097.
2. Yang, Y.; Zhao, L.; Shen, K.; Liu, Y.; Zhao, X.; Wu, Y.; Wang, Y.; Ran, F. Ultra-small vanadium nitride quantum dots embedded in porous carbon as high performance electrode materials for capacitive energy storage. *J. Power Sources* **2016**, *333*, 61-71.
3. Achour, A.; Lucio-Porto, R.; Solaymani, S.; Islam, M.; Ahmad, I.; Brousse, T. Reactive sputtering of vanadium nitride thin films as pseudo-capacitor electrodes for high areal capacitance and cyclic stability. *J. Mater. Sci.: Mater. Electron.* **2018**, *29*, 13125-13131.
4. Guo, J.; Zhang, Q.; Sun, J.; Li, C.; Zhao, J.; Zhou, Z.; He, B.; Wang, X.; Man, P.; Li, Q., *et al.* Direct growth of vanadium nitride nanosheets on carbon nanotube fibers as novel negative electrodes for high-energy-density wearable fiber-shaped asymmetric supercapacitors. *J. Power Sources* **2018**, *382*, 122-127.
5. Lu, X.; Yu, M.; Zhai, T.; Wang, G.; Xie, S.; Liu, T.; Liang, C.; Tong, Y.; Li, Y. High energy density asymmetric quasi-solid-state supercapacitor based on porous vanadium nitride nanowire anode. *Nano Lett.* **2013**, *13*, 2628-2633.
6. Zhang, D.; Li, J.; Su, Z.; Hu, S.; Li, H.; Yan, Y. Electrospun polyporous VN nanofibers for symmetric all-solid-state supercapacitors. *J. Adv. Ceram.* **2018**, *7*, 246-255.

7. Gao, B.; Li, X.; Guo, X.; Zhang, X.; Peng, X.; Wang, L.; Fu, J.; Chu, P.K.; Huo, K. Nitrogen-doped carbon encapsulated mesoporous vanadium nitride nanowires as self-supported electrodes for flexible all-solid-state supercapacitors. *Adv. Mater. Interfaces* **2015**, *2*, 1500211.
8. Zhang, Y.; Wang, X.; Zheng, J.; Hu, T.; Liu, X.; Meng, C. Facile synthesis of high-surface vanadium nitride/vanadium sesquioxide/amorphous carbon composite with porous structures as electrode materials for high performance symmetric supercapacitors. *Appl. Surf. Sci.* **2019**, *471*, 842-851.
9. Dubal, D.P.; Abdel-Azeim, S.; Chodankar, N.R.; Han, Y.-K. Molybdenum nitride nanocrystals anchored on phosphorus-incorporated carbon fabric as a negative electrode for high-performance asymmetric pseudocapacitor. *iScience* **2019**, *16*, 50-62.
10. Zhu, C.; Yang, P.; Chao, D.; Wang, X.; Zhang, X.; Chen, S.; Tay, B.K.; Huang, H.; Zhang, H.; Mai, W., *et al.* All metal nitrides solid-state asymmetric supercapacitors. *Adv. Mater.* **2015**, *27*, 4566-4571.
11. Dubal, D.P.; Chodankar, N.R.; Qiao, S. Tungsten nitride nanodots embedded phosphorous modified carbon fabric as flexible and robust electrode for asymmetric pseudocapacitor. *Small* **2019**, *15*, 1804104.
12. Liu, Y.; Xiao, R.; Qiu, Y.; Fang, Y.; Zhang, P. Flexible advanced asymmetric supercapacitors based on titanium nitride-based nanowire electrodes. *Electrochim. Acta* **2016**, *213*, 393-399.
13. Zhai, T.; Xie, S.; Yu, M.; Fang, P.; Liang, C.; Lu, X.; Tong, Y. Oxygen vacancies

enhancing capacitive properties of MnO_2 nanorods for wearable asymmetric supercapacitors. *Nano Energy* **2014**, *8*, 255-263.

14. Fan, H.; Liu, W.; Shen, W. Honeycomb-like composite structure for advanced solid state asymmetric supercapacitors. *Chem. Eng. J.* **2017**, *326*, 518-527.
